# Bilateral Congenital Absence of the Stapes Superstructure in Two Siblings

**DOI:** 10.1155/2014/901672

**Published:** 2014-06-22

**Authors:** Jose Ignacio Undabeitia, José Undabeitia, Laura Cianci, Luis Padilla, Eduardo Petreñas, Antonio Municio

**Affiliations:** ^1^Otolaryngology, Head and Neck Surgery Department, Cruces University Hospital, Plaza de Cruces s/n, Barakaldo, 48903 Bizkaia, Spain; ^2^Ophthalmology, Dermatology and Otolaryngology, University of the Basque Country Medical School, Barrio Sarriena s/n, Leioa, 48940 Bizkaia, Spain; ^3^Neurosurgery Department, Donostia University Hospital, Calle Doctor Begiristain 117, San Sebastián, 20080 Gipuzkoa, Spain

## Abstract

Middle ear ossicle malformations are an uncommon event. Among them, the congenital absence of the stapes is a very rare condition that is seldom described in the literature. We report the cases of two women, aged 19 and 22 , who presented with a long history of conductive deafness. An exploratory tympanotomy was performed and the absence of the stapes superstructure and an abnormal position of the facial nerve could be observed. A bone anchored hearing aid (BAHA) was implanted in both patients with good results. It is believed that stapes agenesis is related to an abnormal development of the facial nerve, which by the 5th to 6th week of gestation would interpose between the otic capsule and the stapes blastema, preventing these structures from contacting. A long history of nonprogressive hearing loss from birth or early childhood is the key to reach a diagnosis. Several treatment options have been described. The authors opted for a hearing aid due to the high risk of facial nerve lesion, with good functional results.

## 1. Introduction

The congenital anomalies of ossicular chain of the middle ear are an uncommon event that can present in a variety of ways [1–4]. Among these malformations, the congenital absence of a stapes (CAS) is a very rare condition that is seldom described in the English scientific literature. It was first reported by Mcaskile and Sullivan in 1955 [5] in two patients with conductive hearing loss. The CAS has been described in association with other head and neck anomalies and syndromes or as an isolated event [1, 3, 6, 7]. CAS may also occur in patients with normal auditory canal and tympanic membrane, due to their different embryological origin, presenting only as a long history of conductive hearing loss and identified only after surgical exploration [1, 2, 6, 8]. The otologic surgeon should be able to identify these ossicular chain malformations and to understand the embryologic origin of the different structures of the middle ear in order to safely and effectively treat this condition.

## 2. Case Report

**Figure 1:**
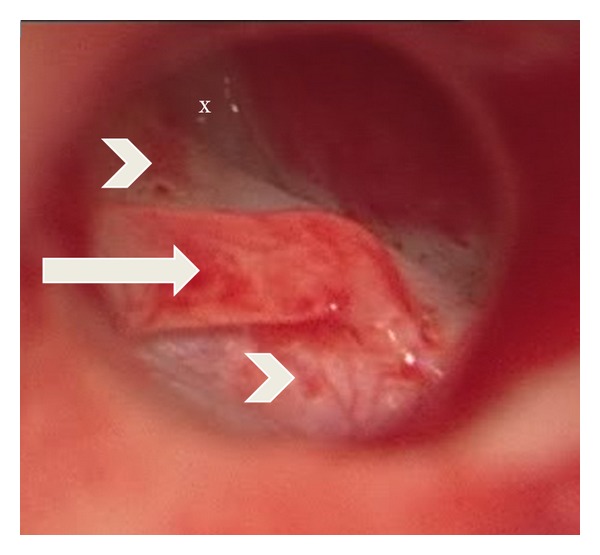
Right exploratory tympanotomy showing the incus (arrow) resting over the facial nerve (arrowhead), a hypoplastic oval window (x), and the complete absence of the stapes superstructure.

We report the case of two sisters of Brazilian origin, aged 19 and 22, who presented with a long history of nonprogressive hearing loss. There was no familiar history suggestive of congenital otologic anomalies. Both patients were products of uneventful pregnancies and deliveries with no history of previous infections, trauma, or related illnesses. The external ear and eardrum were normal on both sides. The audiogram showed bilateral conductive hearing loss for all studied frequencies (250 Hz–4000 Hz) with an average air-bone gap of 60 dB in both patients. Rinne tests were negative bilaterally and the result of Weber's test was central. With an initial suspicion of otosclerosis, a right exploratory tympanostomy was performed to examine the ossicular chain. After careful inspection of the middle ear, we noted the complete absence of the stapes and the stapedial tendon with an abnormal positioning of the facial nerve, which appeared anterior and inferior to the tympanic cavity, above a hypoplastic oval window (Figure 1). Due to this unexpected finding, surgery was finished at this point without any attempt of reconstruction.

**Figure 2:**
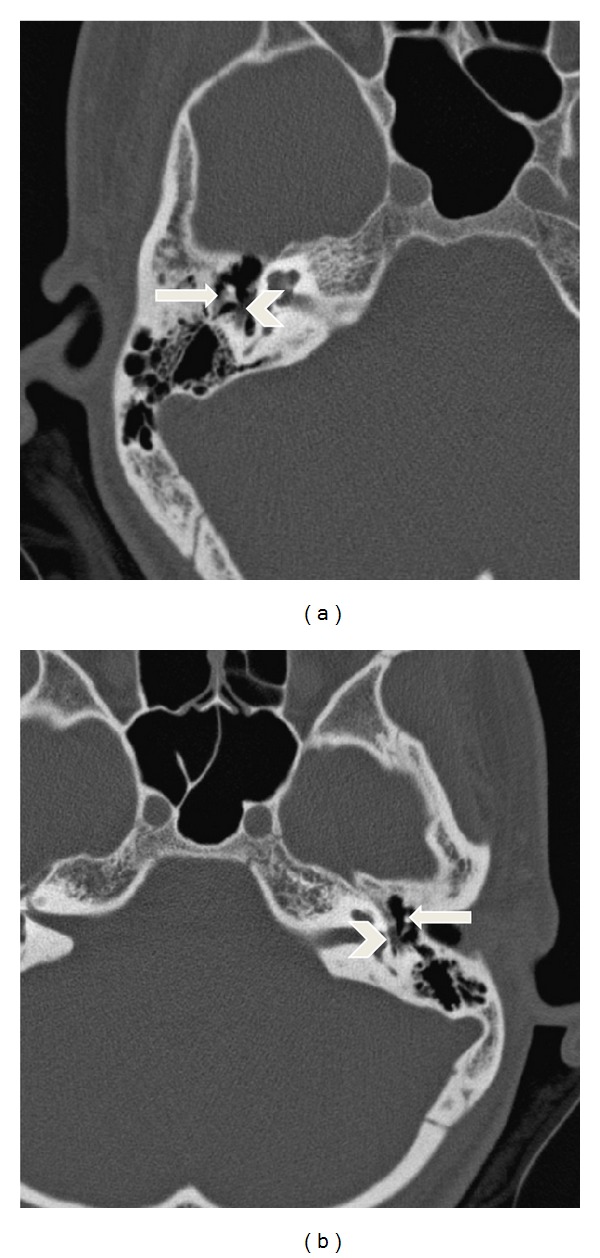
Right (a) and left (b) axial CT scan of the temporal bone showing the bilateral absence of the stapes (arrow), the abnormal position of the facial nerve (arrowhead), normal pneumatization of the mastoid air cells, and normal inner ear morphology.

Due to the similarity of both cases, high resolution temporal bone CT scans were performed, showing the bilateral absence of the stapes superstrucure and the abnormal positioning of the facial nerve and the incus (Figure 2).

Ossicular chain reconstruction was discarded, due to the location of the facial nerve and the high risk of lesion during the procedures. The authors decided to implant a right bone anchored hearing aid (BAHA) in both patients with good functional results. The patients were then referred to the genetic department of our hospital, but no genetic syndrome could be identified.

## 3. Discussion

CAS is a very infrequent condition, seldom described in the literature, first described by Mcaskile and Sullivan in 1955 [5]. It can present as an isolated malformation or associated to other anomalies. The most common of these is the absence or hypoplastic oval window, as in our case. The exact etiology of the CAS is still not known [6, 8]. It has been suggested that the malformation or agenesis of the stapes and oval window is related to the abnormal development of the facial nerve [4, 7, 8]. Regarding the embryologic origin, the malleus head and the incus body differentiate from the fist branchial arch while the malleus handle, long process of the incus, and the stapes develop from the second branchial arch or Reichert's cartilage [1–4, 6, 9, 10]. By the 5th to 6th week of gestation the mass of the stapes becomes recognizable and begins its growth towards the otic capsule [6]. At this same time, both the horizontal and vertical portions of the facial nerve can be identified. If anterior displacement of the facial nerve occurs, it could become interposed between the otic capsule and the stapes blastema, preventing these structures from contacting. This contact is considered essential for the stapes suprastructure development [1, 6, 8]. It has been hypothesized that the displacement of the facial nerve could occur due to a delay in the development of the first branchial arch. This delay would cause a compensatory overshifting of the second branchial arch placing the facial nerve in a more anterior position [6].

A long history of nonprogressive conductive hearing loss since birth or early childhood, with no history of trauma or infection, is highly suggestive of a congenital middle ear malformation. Differential diagnosis should include otosclerosis and acquired traumatic or infectious disruption of the ossicular chain. Also, in the congenital cases, the hearing loss is usually more severe than in the acquired cases [2, 7, 8]. A flat pure-tone audiogram in conversational frequencies, with preserved bone conduction, is also a typical finding. The presence of Carhart's notch in patients with CAS has been described but is not pathognomonic of this condition nor can it always be found, as in our cases [10]. In those patients suspected to suffer from congenital middle ear malformations, high resolution 1 mm section thickness temporal bone CT scan is indicated [2]. However, due to its low incidence, the diagnosis of this condition is challenging and in most instances it can only be reached after direct surgical inspection, as in the cases discussed here.

Due to the lack of published series, management of the CAS remains controversial [6]. Several treatment options have been described, including vestibulotomy and ossicular reconstruction, fenestration of the horizontal semicircular canal, and hearing aids [7, 8]. In the author's opinion, the treatment of choice is the ossicular chain reconstruction when feasible. This reconstruction can be complicated if the facial nerve is interposed between the remaining ossicles and the oval window, since the facial nerve usually overlies the oval window. If necessary, the facial nerve can be retracted inferiorly or superiorly; however, this manipulation can result in a higher risk of nerve lesion [6, 7]. In our patients, due to the bilateral presence of the malformation and the localization of the facial nerve, the authors opted for a bone anchored hearing aid (BAHA), which provided good functional results. Ideally, treatment should be individualized to each patient, according to the anatomic findings and associated malformations.
